# The Biological Role of Klotho Protein in the Development of Cardiovascular Diseases

**DOI:** 10.1155/2018/5171945

**Published:** 2018-12-24

**Authors:** Agnieszka Olejnik, Aleksandra Franczak, Anna Krzywonos-Zawadzka, Marta Kałużna-Oleksy, Iwona Bil-Lula

**Affiliations:** ^1^Department of Medical Laboratory Diagnostics, Division of Clinical Chemistry, Wroclaw Medical University, 50-556 Wroclaw, Poland; ^2^Department of Cardiology, University Hospital of Lord's Transfiguration, Poznan University of Medical Sciences, 61-848 Poznan, Poland

## Abstract

Klotho is a membrane-bound or soluble antiaging protein, whose protective activity is essential for a proper function of many organs. In 1997, an accidental insertion of a transgene led to creation of transgenic mice with several age-related disorders. In Klotho-deficient mice, the inherited phenotypes closely resemble human aging, while in an animal model of Klotho overexpression, the lifespan is extended. Klotho protein is detected mainly in the kidneys and brain. It is a coreceptor for fibroblast growth factor and hence is involved in maintaining endocrine system homeostasis. Furthermore, an inhibition of insulin/insulin-like growth factor-1 signaling pathway by Klotho regulates oxidative stress and reduces cell death. The association between serum Klotho and the classic risk factors, as well as the clinical history of cardiovascular disease, was also shown. There are a lot of evidences that Klotho deficiency correlates with the occurrence and development of coronary artery disease, atherosclerosis, myocardial infarction, and left ventricular hypertrophy. Therefore, an involvement of Klotho in the signaling pathways and in regulation of a proper cell metabolism could be a crucial factor in the cardiac and vascular protection. It is also well established that Klotho protein enhances the antioxidative response via augmented production of superoxide dismutase and reduced generation of reactive oxygen species. Recent studies have proven an expression of Klotho in cardiomyocytes and its increased expression in stress-related heart injury. Thus, the antioxidative and antiapoptotic activity of Klotho could be considered as the novel protective factor in cardiovascular disease and heart injury.

## 1. Introduction

The leading cause of death among Europeans and around the world is cardiovascular disease (CVD) [1]. There are well-established risk factors for cardiovascular damage and coronary artery disease such as diabetes, dyslipidemia, hypertension, and obesity. However, the susceptibility, severity, and progression of heart disorders are not fully understood [2]. Given importance to assess and control pathological process in heart tissue, focusing on finding new markers and treatment of myocardial damage seems to be necessary.

Klotho protein is associated with aging and the name of gene comes from Greek mythology [3]. Clotho, one of the Three Fates, was responsible for spinning the thread of human life. In recent years, there is quite a lot of interest in Klotho protein and some diseases. Scientists are trying to define the function of Klotho and its role in the dysfunction of many organs. Understanding the mechanism of Klotho protection can provide information on the most prevalent disorders, such as cardiovascular disease.

## 2. The Structure of Klotho Protein

*Klotho *(*kl*) gene spans approximately 50 kbp and is composed of 5 exons. Its two transcripts encode membrane and secreted proteins [4].* Kl* gene highly correlates with the suppression of several ageing phenotypes [3, 5]. It has been identified for the first time in 1997, when Kuro-o et al. showed that an insertion of a transgene led to a syndrome resembling ageing in Klotho-deficient mice (*kl-/kl-*) [3]. The insertional mutation was located in the 5' flanking region and resulted in an 8 kbp deletion, 6 kbp upstream of a transcription start site. Therefore, the coding structure of* kl* gene was not disrupted, but the expression was reduced and the loss of Klotho mRNA was observed [3, 4]. The disturbances of* kl* gene expression in mice were accompanied by a short lifespan, infertility, and several age-related disorders such as atherosclerosis, osteoporosis, age-related skin changes, and ectopic calcifications [3, 4]. At the age of 3 to 4 weeks, the animal models show growth retardation and they usually die at 8–9 weeks of age [3]. The expression of* kl* gene is observed mainly in the kidneys and brain, as well as in the pituitary gland, placenta, skeletal muscle, urinary bladder, aorta, pancreas, testis, ovary, colon, and thyroid gland [3, 4, 6].

*Kl* gene encodes a single-pass transmembrane glycoprotein type 1 (1014 and 1012 amino acids in both mouse and human), which is expressed in the cell membranes and Golgi apparatus [4, 7]. The molecular weight of Klotho is 135 kDa and its value is influenced by N-glycosylation [8–11]. Two internal repeats (termed mKL1 and mKL2), about 450 amino acids long each, create the extracellular domain which shows a high homology to the family 1 of *β*-glucosidases. Those glucosidases hydrolyze a *β*-glycosidic linkage in the saccharides, glycoproteins, and glycolipids [3, 7, 12].

**Figure 1:**
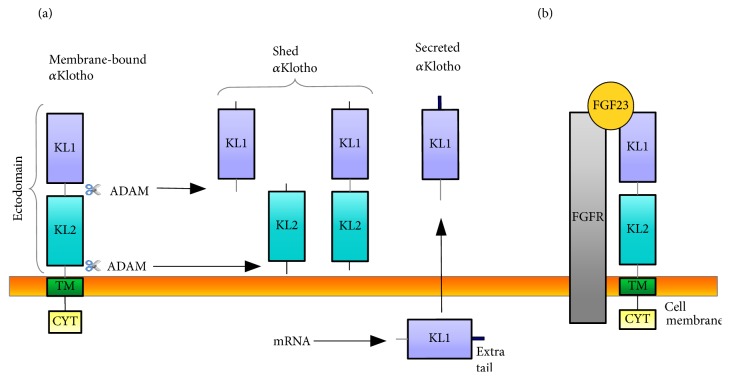
The scheme of membrane-bound and soluble (shed and secreted) forms of *α*Klotho. (a) Membrane-bound *α*Klotho is created by 3 domains: cytoplasmic (CYT), transmembrane (TM), and ectodomain. The ectodomain has two internal repeats, KL1 and KL2. Membrane-bound *α*Klotho is subjected to shedding of ectodomain by ADAM 10 or 17 protease in two ways to release three shed *α*Klotho. The alternative transcriptional termination of* kl* gene expression leads to generation of secreted *α*Klotho. (b) Membrane-bound *α*Klotho forms a complex with FGFR to create a high-affinity binding site for FGF23. ADAM, a disintegrin and metalloproteinase domain-containing protein; FGFR, fibroblast growth factor receptor; FGF23, fibroblast growth factor 23.

The main type of Klotho protein is called *α*Klotho (Klotho) (Figure 1(a)). There are two types of soluble *α*Klotho protein: shed and secreted. Three forms of shed *α*Klotho are formed as a consequence of the membrane proteases ADAM10 and ADAM17 (a disintegrin and metalloproteinase domain-containing proteins 10 and 17) activity. They cleave Klotho fragments from the extracellular domain of membrane-bound Klotho into the blood, urine, and cerebrospinal fluid [7, 12–14]. ADAM10 and 17 cut the Klotho in two ways to generate the molecules of 130 kDa (the major product of shedding) and 68 kDa, respectively [8, 15]. First cut occurs just behind the transmembrane domain and the product (130 kDa) contains both KL1 and KL2 fragments. Second cut is localized within the connecting segment of KL1 and KL2 domains and releases protein (68 kDa) containing only KL1 domain [8, 12, 13]. When the cleavage occurs in both sides, KL1 and KL2 domains are released separately [12]. Additionally, the alternative transcriptional termination of* kl* gene leads to the generation of secreted Klotho (about 65 kDa). The transcript contains 3 exons in mice (lacking exons 4 and 5) and 5 exons in humans [4, 5, 15].* KL* gene transcript of secreted form of Klotho protein predominates over the membrane form in human [5]. This form of Klotho protein includes 549 amino acid residues, corresponds to the KL1 fragment, and has an additional C-terminal sequence [4, 7, 12, 13]. It is found in the blood, urine, and cerebrospinal fluid and its level declines with age [4, 16].

Based on the sequence similarity, two other Klotho-related genes which encode *β*-Klotho and *γ*-Klotho proteins have been described. *β*-Klotho and *γ*-Klotho are the single-pass transmembrane glycoproteins type 1 likewise [7, 14, 17]. It was demonstrated that *β*-Klotho is composed of KL1 and KL2 domains, shares 41% amino acid identity with *α*-Klotho, and is primarily expressed in the liver, gastrointestinal tract, spleen, kidney, and adipose tissue. *γ*-Klotho is composed of KL1 domain only and can be found mainly in the adipose tissue, eyes, and kidneys [2, 7, 14].

It should be noted that there are some difficulties in the measurement of soluble Klotho in serum or urine. In 2010, Yamazaki et al. tested antibodies specifically recognizing an extracellular domain of human serum *α*Klotho. As a result, they established ELISA test for detection of circulating soluble *α*Klotho protein for the first time [18]. However, due to the highly conserved sequences between different Klotho forms, it is difficult to differentiate full-length soluble *α*-Klotho from other short forms (KL1 or KL2 domains) and secreted Klotho, present in the body fluids. Likewise, in 2000, Kato et al. established the anti-Klotho monoclonal antibodies, KM2119, with recognition site for KL2 domain, and KM2076 and KM2365 with recognition site for KL1 domain [19]. Nevertheless, using currently available antibodies or commercial tests for the quantitative measurement of *α*Klotho in the body fluids, it is possible to measure only total soluble *α*Klotho. It is difficult to characterize detected forms of *α*Klotho (full-length Klotho, KL1 or KL2 fragments). Moreover, it is demanding to make a distinction between shed or secreted Klotho. A specific antibody against the variant peptide fragment may help resolve this problem, for example, polyclonal antibody against an additional C-terminal sequence in measurement of secreted Klotho. We expect technical advances will allow these key details to be filled in the future.

## 3. The Biological Functions of Klotho Protein

The membrane-bound Klotho protein is needed to increase an affinity of endocrine fibroblast growth factor (FGF) to FGF receptors (FGFR) at their target organs. Twenty-two members of FGFs have been identified, a large superfamily of peptides which impact a wide range of biological processes in human. FGFs bind to FGFRs to form a complex which functions as a paracrine/autocrine factor [14]. FGF19, FGF21, and FGF23 are classified as a FGF19 subfamily and act as the endocrine factors or hormones in the regulation of energy and mineral metabolism [14].

The main function of membrane-bound Klotho is to form a complex with FGFR1c, FGFR3c, and FGFR4 and to convert their canonical forms into the specific receptors for FGF23, a bone-derived phosphaturic hormone (Figure 1(b)) [7, 14, 17]. It is well known that FGF23 inhibits the phosphate transporters NaP_i_-IIa and downregulates the reabsorption of inorganic phosphate (P_i_) in the renal proximal tubules [7, 14, 20]. In the renal distal tubules, FGF23 stimulates the reabsorption of calcium (Ca^2+^)* via* transient receptor potential vallinoid-5 channel (TRPV5) and regulates Ca^2+^ homeostasis [7, 21]. Moreover, FGF23 downregulates the expression of 1*α*-hydroxylase (CYP27B1) in the renal proximal tubules and consequently suppresses the synthesis of biologically active 1,25-dihydroxyvitamin D_3_ (calcitriol). Calcitriol is responsible for absorption of intestinal and renal phosphate and calcium. An elevated level of calcitriol in* kl-/kl-* mice led to increased plasma Ca^2+^ and P_i_ levels, as well as to vascular calcification [20, 22]. Hence, FGF23 induces a negative phosphate and calcium balance by promoting their excretion into urine and by inhibition of their absorption in the intestine [7, 14, 20, 22]. It should be mentioned that the parathyroid gland presents high expression of Klotho and is another target for FGF23. Studies have established clearly that FGF23 reduces the secretion of parathyroid hormone, which is involved in the maintaining of phosphate and calcium balance [23]. For this reason, membrane-bound Klotho acts as an obligatory coreceptor for FGF23, thus regulating mineral metabolism [7, 17].

It needs to be pointed that soluble form of Klotho can maintain the ion homeostasis independently of FGF23. Klotho shows the *β*-glucuronidase and sialidase activity and modifies the function of renal and intestinal transporters [7, 24, 25]. The reabsorption of renal Ca^2+^ and secretion of potassium (K^+^) are regulated by TRPV5 and renal outer medullary potassium channel 1 (ROMK1), respectively. TRVP5 is found predominantly along the distal tubules and ROMK1 is expressed in the distal and collecting tubules [26–29]. It is known that N-glycans are covalently attached to protein at asparagine residues, and sialic acid (N-acetylneuraminic acid) can be attached to the terminal galactose of N-glycans by *α*2,3, *α*2,6, or *α*2,8 linkage [30, 31]. It has been reported that soluble Klotho removes terminal *α*2,6-linked sialic acids from TRPV5 and ROMK1 N-glycan chains and uncovers the underlying disaccharide, galactose-N-acetyloglucosamine (LacNAc). LacNAc is a ligand for galactose-binding lectin, galectin-1. It is crucial that galectin-1 binds LacNAc and *α*2,3-sialylated LacNAc, but not *α*2,6-sialylated LacNAc. Therefore, removal of terminal *α*2,6-linked sialic acid (but not 2,3-linked sialic acid) from N-glycans of TRPV5 and ROMK1 by Klotho exposes underlying LacNAc for binding to galectin-1 on the extracellular side of cell membrane. Then, the channels are accumulated on the plasma membrane because of binding to galectin-1 lattice at the extracellular surface. It prevents the TRPV5 and ROMK1 endocytosis. Consequently, the abundance of cell-surface TRPV5 and ROMK1 is increasing, and the renal reabsorption of calcium and the secretion of potassium into urine are enhanced [32–37]. It was found that full-length soluble Klotho, as well as purified KL1 and KL2 domains, each can regulate ROMK1 [32]. Moreover, high K^+^ intake increases the activity of ROMK1 in the apical membrane of cortical collecting duct cells, to secrete more K^+^ into the urine and maintain K^+^ balance. Experimental data showed a defect in secretion of K^+^ and hyperkalemia in* Romk1*^−/−^ mice under high K^+^ diet [28]. The regulation of K^+^ homeostasis by Klotho was examined by Cha et al. (2009). The intravenous administration of soluble Klotho in rats increased urinary K^+^ excretion. It was due to enhanced net tubular secretion of K^+^. They found that soluble Klotho appeared in urine, which supports the thesis that Klotho regulates ion channels from the luminal side [32]. Surprisingly, Klotho administration did not alter the plasma K^+^ level [32]. It may be because Klotho increases resistance to insulin on the peripheral tissues, which leads to increase in extracellular K^+^ [32, 38–40]. However, the association between Klotho, ROMK1, and plasma K^+^ level needs to be further investigated. It also remains unclear how injected Klotho reaches urinary space. Hu et al. (2016) proposed that Klotho is expressed in the kidney, released into the systemic circulation by secretases-mediated shedding, and cleared from blood through the kidney into the urine. They showed that Klotho traffics across renal proximal tubules by transcytosis and is secreted into the urinary lumen [41]. Other possibilities include shedding of membrane Klotho expressed in the apical membrane of tubular cells [32]. It should be also mentioned that soluble Klotho can act as a phosphaturic factor and inhibits phosphate transporters NaP_i_-IIa in the renal proximal tubules. Through its *β*-glucuronidase activity, Klotho modifies NaP_i_-IIa glycans and leads to their internalization from the apical membrane leading to decreased absorption of phosphates, also independently of FGF23 [7, 42].

*β*Klotho, the another Klotho family member, is a coreceptor for FGF15/19 and FGF21 and influences energy metabolism [7, 43]. Postprandial, bile acids induce the expression of FGF15/19 in the intestine and then FGF15/19 interacts with *β*Klotho and FGFR4 in the liver. In consequence, the gluconeogenesis and synthesis of bile acids are suppressed (negative feedback loop), while the production of hepatic glycogen and proteins is increased [7, 14]. By contrast, FGF21 is secreted from the liver upon fasting state and creates a complex with *β*Klotho and FGFR1c in the white adipose tissue and consequently promotes the lipolysis. FGF21 also stimulates a glucose uptake in the adipocytes and induces an insulin synthesis in the pancreatic *β* cells [7, 14, 43–45]. The recent investigation of Lan et al. (2017) reported that neuronal *β*-Klotho is necessary for FGF19 and FGF21 to regulate weight loss and glycemia [46]. Based on the report, *β*Klotho, FGF19, and FGF21 activate the sympathetic nervous system and then metabolism of the brown adipose tissue. Therefore, the enhanced insulin sensitivity and thermogenesis are observed [46].

## 4. The Role of Klotho Protein in Oxidative Stress and Cell Apoptosis

Soluble form of Klotho is recognized as having the activity of endocrine, autocrine, and paracrine hormone. It has been shown that Klotho is involved in the regulation of oxidative stress, inflammation, and fibrosis by the inhibition of insulin/insulin-like growth factor-1 (IGF-1) and transforming growth factor-*β*1 (TGF-*β*1) signaling pathways [7, 11, 37, 47–49].

It is well documented that the forkhead box protein O transcription factors (FoxOs) are suppressed through phosphorylation by the phosphatidylinositol 3-kinase (PI3K)/serine-threonine kinase (Akt) signaling pathway. FoxOs control genes which are involved in the cellular differentiation, growth and survival, cell cycle, glucose and lipid metabolism, and detoxification of reactive oxygen species (ROS) [37, 50]. The suppression of insulin/IGF-1/PI3K signaling pathway leads to activation of FoxOs and enhanced expression of manganese superoxide dismutase (MnSOD), superoxide neutralizer. Consequently, the inhibition of insulin/IGF-1/PI3K signaling by Klotho results in an oxidative stress resistance and contributes to an antiaging mechanism [37, 47, 50]. More recently, Takenaka et al. (2017) revealed an alleviation of blood pressure, albuminuria, and oxidative stress in diabetes after the supplementation of exogenous Klotho protein in mice [51]. The authors also proposed an administration of recombinant Klotho to inhibit insulin/IGF-1 signaling pathway and to affect diabetic nephropathy [51].

In 2017 Lim et al. analyzed the tacrolimus-induced oxidative stress in mice [52]. They demonstrated the functional and histological improvements of renal tissue after Klotho administration. Moreover, mitochondrial production of ROS and mitochondrial dysfunction caused by tacrolimus were diminished by means of Klotho. Data has proven that Klotho negatively regulated the PI3K/Akt pathway and subsequently enhanced FoxO-mediated expression of MnSOD. Finally, Klotho led to diminution of oxidative stress in mice [52]. Similarly, the antioxidative action of Klotho was notified in a model of paraquat treated HeLa cells [47]. Paraquat is a kind of herbicides which generates superoxide production. The results showed the abolished apoptosis and attenuated oxidation of lipids in living cells after their incubation with soluble recombinant Klotho [47]. Moreover, urinary level of 8-hydroxy-2′-deoxyguanosine (a biological marker of* in vivo* oxidative DNA damage) of long-lived Klotho-overexpressing transgenic mice was reduced. It suggests the Klotho-induced prevention of oxidative DNA damage [47].

There are speculations that expression of Klotho is decreased under the sustained stress conditions [49, 53]. The suppression of* klotho *gene was disclosed in the study of mouse inner medulla collecting duct 3 (mIMCD3) cells that have undergone an oxidative stress. An oxidation was caused by an exposure to hydrogen peroxide (H_2_O_2_). Surprisingly, an insertion of exogenous* kl* gene into mIMCD3 cells resulted in the significantly diminished H_2_O_2_-induced apoptosis. This confirms that Klotho may protect cells from death caused by oxidative stress [53]. Similar remarks were proclaimed in a rat model of ischemia-reperfusion (I/R) kidney injury. The ischemic acute renal failure was exacerbated through the downregulation of renal Klotho, whereas an induction of* kl* gene expression protected tissue from I/R injury [54]. Similarly, in 2015 Oh et al. observed patients with end-stage renal disease (ESRD) and showed that Klotho deficiency was related to an enhanced oxidative stress and inflammation in ESRD [55]. Therefore, taking into account the fact that the development of cardiovascular disease in ESRD patients is common, Klotho protein could provide the protection of vasculature and cardiac tissue [55].

## 5. *KLOTHO* Gene Polymorphism

More than 10 mutations or single nucleotide polymorphisms (SNPs) in human* KLOTHO *(*KL*) gene were recently shown. They are characterized by their large degree of pleiotropic associations, especially with the kidney diseases, coronary artery disease, stroke, and bone mineral density [56, 57].

*KL-VS*, an allele of* KLOTHO *gene, is a common haplotype in general population. It was defined by the presence of 6 SNPs in exon 2 and flanking sequence. Three mutations of* KL* gene in exon 2 include two amino acid substitutions (F352V, C370S) and one silent mutation (K385K).* KL-VS* allele affects the metabolism and activity of Klotho, contributes to the human age-related phenotypes, and is connected with a reduced lifespan in homozygosity [58–62]. It also influences an intelligence and cognition in human [59, 60]. It is worthy to mention that* KL-VS* could be a marker of enhanced risk for the development of cardiovascular diseases [62, 63]. The investigators emphasize the relevance of this polymorphism on an occurrence of early-onset coronary artery disease (CAD). Moreover,* KL-VS* is related to an enhanced level of serum high-density lipoprotein, systolic blood pressure, and an augmented risk of stroke. This supports the hypothesis of Klotho association with atherosclerosis [62, 63]. In addition, data affirmed the relationship between rs650439 (intron 4), the other representative single nucleotide polymorphism of* KL* gene, and carotid atherosclerosis in hypertensive patients [64].

The single nucleotide polymorphisms G395A located in the promotor and C1818T in exon 4 are other, well-studied, variants of human* KL* gene. They are recognized as being associated with hypercalcemia, hypophosphatemia, phosphaturia, and low bone mineral density [60, 65, 66]. It has been established that G395A polymorphism can be an independent risk factor for the development of coronary artery disease [67]. Interestingly, more frequent presence of G395A allele did not occur in patients with vasospastic angina, indicating that the polymorphism may be associated with CAD due to atherosclerosis [67]. Similarly, there was an association between the variant of the* KL* gene and cardiovascular risk factors in an examination of healthy Korean females [68]. The analysis revealed higher mean systolic blood pressure in carriers of G395A and higher plasma glucose level in carriers of C1818T allele which constitutes an increased peril of CVD [68]. Moreover, the recent study of Elghoroury et al. (2018) proclaimed the link between G395A polymorphism and cardiovascular complications [56]. It is known that end-stage renal disease (ESRD) results in the several cardiac disorders. The research group included the pediatric patients with chronic kidney disease (CKD), in which cardiovascular disease was studied in relation to the prevalence of left ventricular hypertrophy (LVH), severe LVH, and dilated cardiomyopathy [56]. The outcomes disclosed significantly higher frequency of A allele of G395A in ESRD patient with LVH [56]. Substantially, these findings affirmed the relevance of G395A polymorphism on the development of ESRD and CVD [56].

To conclude, the presented data showed the importance of* KLOTHO* gene polymorphisms and occurrence of atherosclerosis, coronary artery disease, or left ventricular hypertrophy. Thus, the allele A of* KL *gene could be considered as the cardiovascular risk factors.

## 6. Klotho Deficiency and Risk for Cardiovascular Disease

**Figure 2:**
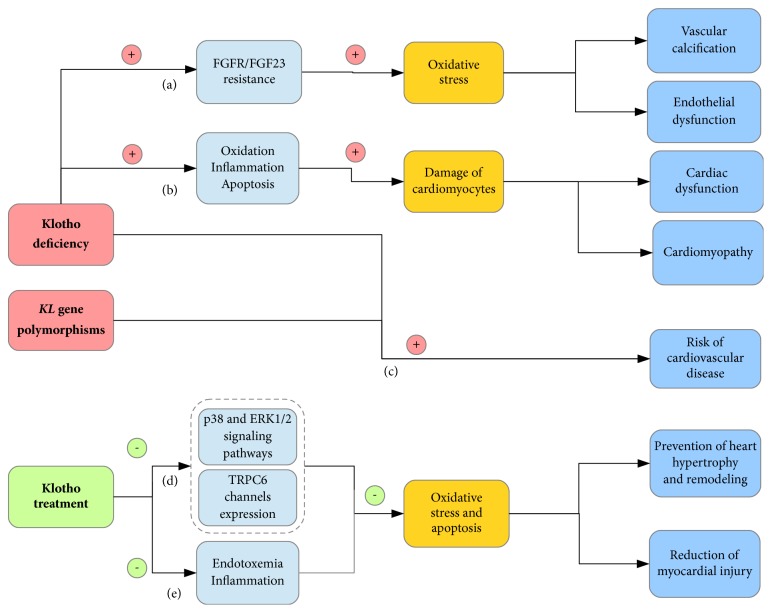
The scheme of an expected mechanism by which Klotho protein is involved in cardiovascular diseases. (a) Local deficiency of vascular-derived Klotho leads to calcification. It is related to the FGFR/FGF23 resistance, which in turn inhibits the anticalcific effect of FGF23. An attenuated expression of Klotho protein in vessel wall reduces production of NO and increases formation of ROS. Therefore, an imbalance of Klotho and FGF23 leads to oxidative stress and endothelial dysfunction. (b) Depletion of Klotho can promote the prooxidative, proinflammatory, proapoptotic activity and damage of cardiomyocytes in the state of CVD risk. As a consequence, cardiac dysfunction and cardiomyopathy may be observed. (c) Klotho deficiency and* KL* gene polymorphisms are the risk factors for cardiovascular disease and correlate with the development of atherosclerosis, CAD, MI, or LVH. (d) An occurrence of cardiac hypertrophy and remodeling in the state of Klotho deficiency is related to oxidative stress. It is caused by the activation of p38 and ERK1/2 signaling pathways, as well as by the overexpression of TRPC6 channels in heart. The treatment with exogenous Klotho may provide protection against the fibrotic alterations. (e) Klotho contributes to alleviation of cardiac dysfunction and pathological changes in toxemic and ischemic heart. The treatment with Klotho mitigates an inflammation, ROS generation, apoptosis, mitochondrial dysfunction, fibrosis, and hypertrophy. Klotho may induce the restoration of cardiac function and thus could be explored as a therapeutic factor in myocardial injury. FGFR, fibroblast growth factor receptor; FGF23, fibroblast growth factor 23; NO, nitric oxide; ROS, reactive oxygen species; CAD, coronary artery disease; MI, myocardial infarction; LVH, left ventricular hypertrophy; ERK1/2, extracellular signal-regulated kinase 1/2; TRPC6, transient receptor potential canonical 6; 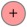, induction; 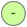, reduction.

Taking into account the fact that Klotho is involved in a resistance to oxidative stress, some scientists have focused on the role of Klotho in the development of cardiovascular diseases (Figure 2).

The first investigation of plasma Klotho in the aspect of CVD was performed in 2011 by Semba et al. [69]. The analysis included the common cardiovascular risk factors such as age, sex, total cholesterol, HDL cholesterol, systolic blood pressure, and diabetes. Interestingly, the risk of CVD in adults with higher plasma Klotho was lower [69]. Similarly, there was an association between plasma Klotho and CVD in fifty healthy volunteers without any known risk factors for cardiovascular disorders [70]. Early predictors of atherosclerosis such as the thickness of carotid artery intima-media, flow-mediated dilation of the brachial artery, and the thickness of epicardial fat were explored [70]. The results showed that low serum Klotho level was associated with larger thickness of epicardial fat and carotid artery intima-media and lower flow-mediated dilation of the brachial artery. Thus, lower levels of serum Klotho should be considered as an early predictor of atherosclerosis [70].

Based on the previous studies proclaiming a potentially positive effect of Klotho in CVD, in 2016 Corsetti et al. assessed that Klotho is expressed in myocardial tissue and proved its association with the prevalence of CVD [2]. The right atrium biopsy samples of thirty patients at high and low risk for atherosclerotic cardiovascular disease (ASCVD) were examined. Research showed that Klotho, *β*-Klotho, FGF21, and FGF23 proteins are expressed in cardiomyocytes and subjects at higher cardiovascular risk had a reduced expression of cardiac Klotho and an elevated expression of cardiac FGFs [2]. The analysis of cardiomyocytes from patients at high risk for ASCVD also revealed an augmented expression of glucose-regulated protein 78 (GRP78), a marker of endoplasmic reticulum (ER) stress, and superoxide dismutase 1 (SOD1), an oxygen free radicals scavenger [2]. It is also known that high level of nitric oxide (NO) triggers an inflammation in cardiac tissue and disrupts its contractile function [71]. An expression of inducible nitric oxide synthase (iNOS) and subsequent NO formation can be induced by the agents such as inflammatory cytokines and endotoxins. It is also mediated through cytokine-inducible transcription factors like nuclear factor-kappa-B (NF-*κ*B), which is accountable for the inflammatory responses to a variety of signals [72–75]. Therefore, an excessive activation of NF-*κ*B and overexpression of iNOS in cardiomyocytes lead to the cellular dysfunction and damage and, hence, result in the cardiomyopathy, bradyarrhythmia, and sudden cardiac death [72–74]. Importantly, the immunoreactivity of these molecules was increased in cardiomyocytes obtained from subjects at high risk for ASCVD, confirming the active inflammation and apoptosis of these cells [2]. On the contrary, endothelial nitric oxide synthase (eNOS) is expressed constitutively and participates in the vasodilation, blood pressure, vasoprotection, and antiatherosclerotic action [72, 73]. An expression of cytoplasmic eNOS in cardiomyocytes of patients at high risk for ASCVD was significantly decreased and hence led to disruption of cellular energetic metabolisms and mitochondrial biogenesis [2]. Authors also emphasized higher expression of TGF-*β*1 intensified fibrosis [2]. On this basis, there is a deep suggestion that depletion of Klotho can promote prooxidative, proinflammatory, and proapoptotic activity in cardiomyocytes, leading to their damage in patients at higher risk of cardiovascular diseases. Thus, expression of cardiac Klotho might improve the ability of cells to withstand the stress conditions [2].

There is a link between Klotho deficiency and CKD, renal disorders, and kidney damage. Several studies mentioned that Klotho can protect the kidneys against decline of their function [76, 77]. In the research including patients with CKD undergoing hemodialysis, an expression of Klotho was significantly reduced. It should be noted that high serum level of Klotho was associated with the low risk of cardiovascular events and death during chronic dialysis [78]. It has become clear that CKD is associated with the cardiovascular episodes, so the repletion of Klotho synthesis could provide renal protection and thus counteracts the accidents of cardiovascular mortality [78, 79].

Interestingly, Klotho protein was also found in the sinoatrial node pacemaker cells in mice [80]. An* in vivo* research of Klotho-deficient mice (*kl-/kl-*) subjected to restraint stress showed that* kl-/kl-* mice died more frequently than wild-type animals. The sinoatrial conduction block or sinus arrest in* kl-/kl-* mice (shown as bradyarrhythmias) was observed [80]. Therefore, data confirmed that sinoatrial node dysfunction and sudden death were observed in Klotho-deficient mice. Taking into account the previous results and an impact of Klotho deficiency on heart function, it explains that Klotho expression is essential for the proper and robust pacemaking activity of sinoatrial node [80].

The above data showed that Klotho deficiency is independently related to the development of cardiovascular disease and its proper expression could provide the cardiovascular protection. An intracellular distribution of Klotho in myocardium is necessary for appropriate metabolism of cardiomyocytes and heart physiology.

## 7. Protective Role of Klotho Protein in Ischemic Injury

Experimental studies of Klotho protein in ischemic tissues have apparently affirmed its protective role in several animal models. Zhou et al. (2018) investigation of ischemic injury in murine brain proclaimed the neuroprotection and inhibition of neuropathological changes after an induced overexpression of Klotho [81]. Similarly in another research, it has been confirmed that the repletion of Klotho restrained the progression of acute kidney injury (AKI) to CKD in a mouse model of renal I/R injury [82]. Taking into account the fact that kidney disorders are an independent risk factor for the development of CVD, some further studies focused on its association with CVD. In 2017 Hu et al. explored that, in the state of CKD and uremic cardiomyopathy, the treatment with recombinant Klotho may affect prophylactic and therapeutic [77]. The supplementation of Klotho after I/R counteracted the progression of AKI to CKD in a mouse model. Moreover, they noticed that injection of Klotho after the onset of CKD retards the development of disease. It had also an impact on the kidney and heart morphology [77]. The clinicians emphasize that Klotho is associated with reduction of renal fibrosis, cardiac hypertrophy, and remodeling [77]. For these reasons Klotho may serve as a beneficial factor in the kidney diseases and in the uremic cardiomyopathy as well [77, 83].

Klotho has been widely studied due to its protective role in toxemic heart damage [84]. HSP70 is an anti-inflammatory molecule, induced during cell stress to protect tissues from an injury and to affect apoptosis. HSP70 can also interact with NF-*κ*B and block its activation and can modulate the response to inflammatory cytokines [75, 84]. In the study of aging endotoxemic mouse heart, the heart damage and cardiac dysfunction were generated by administration of lipopolysaccharide as a proinflammatory agent. As a result, mice developed endotoxemic left ventricular dysfunction. The injury was more severe and sustained in older animals. Endotoxemia also caused the depletion of cardiac Klotho and HSP70, but administration of recombinant Klotho preserved the myocardial expression of HSP70 and improved the cardiac function in aging subjects. Hence, HSP70 was involved in the anti-inflammatory action of Klotho. It should be also noted that Klotho mitigated the activation of myocardial NF-*κ*B and led to decreased production of proinflammatory mediators. These novel findings suggest that the inflammatory response and disrupted cardiac function are associated with Klotho deficiency. Nevertheless, Klotho may serve as a therapeutic factor in the amelioration of endotoxemic cardiac disturbances related to aging [84].

In 2018, Guo et al. presented a study of cardioprotection by Klotho in a hyperglycemia-induced injury of hearts in diabetic mice, as well as in cells subjected to high glucose [85]. Regarding the diabetes-stimulated mouse heart, an improvement of cardiac function and mitigation of cardiac oxidative stress, decreased cell death, and remodeling after the Klotho injection were observed. Moreover, the treatment of rat ventricular cells (H9c2) and neonatal rat cardiomyocytes with Klotho reduced inflammation, generation of ROS, apoptosis, mitochondrial dysfunction, fibrosis, and hypertrophy. The analysis of proinflammatory factors in these cells showed reduced activation of NF-*κ*B, confirming suppressed inflammatory responses. The antioxidative effect of Klotho was also constituted through the augmented expression of nuclear factor erythroid 2-related factor 2 (Nrf2), which plays a pivotal role in the regulation of oxidative stress [85]. Activation of Nrf2-antioxidant response signaling pathway is crucial in a cellular defense [86]. Considering an induction of apoptosis in the above cells, Klotho pretreatment decreased the activity of caspase-9 and caspase-3 and protected against cell death. This confirms the beneficial influence of Klotho on cell metabolism and survival [85]. For this reason, it is suggested that Klotho could be a therapeutic factor in the development and treatment of diabetic cardiomyopathy [85].

The above findings constitute the importance of Klotho in reduction of cardiac dysfunction and morphological changes. Klotho is highly involved in cell defense against the oxidative stress and may induce the restoration of cardiac function in response to myocardial injury. Klotho could be tested as a potential therapeutic factor in myocardial tissue impairment.

## 8. *β*Klotho as a Coreceptor for Fibroblast Growth Factor 21 in Myocardium

**Figure 3:**
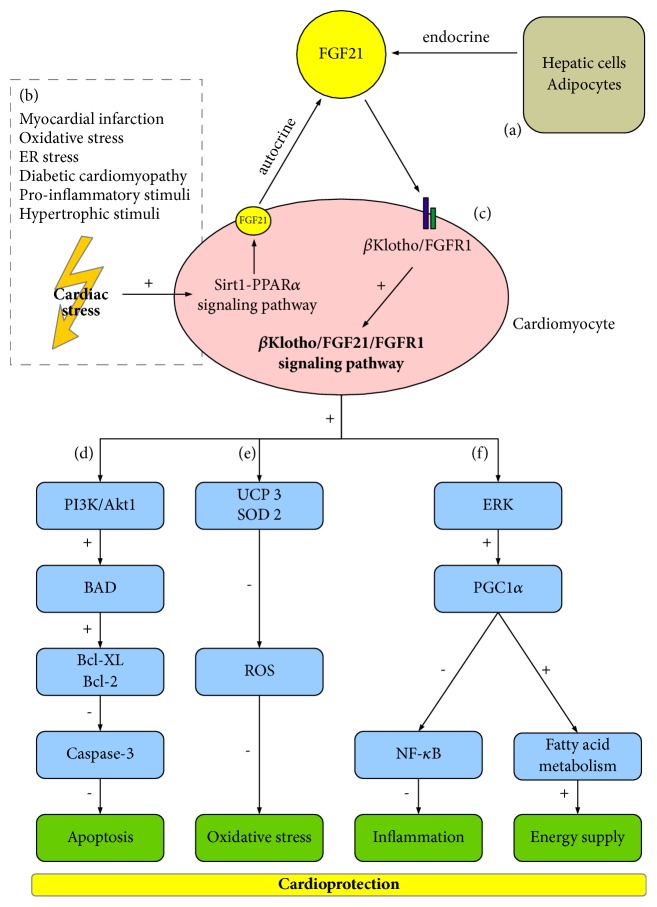
Schematic representation of FGFR1/FGF21/*β*Klotho complex actions in the cardiomyocytes. (a) FGF21 is produced mainly in the liver and adipose tissue. (b) Different cardiac stress stimuli activate Sirt1-PPAR*α* pathway in the heart. It leads to production of FGF21 in the cardiomyocytes in an autocrine manner. (c) FGF21 creates a complex with membrane-bound *β*Klotho and FGFR1 in the cardiac cells. Activation of FGFR1/FGF21/*β*Klotho network induces cardioprotection. (d) The activation of cell survival PI3K/Akt1 pathway leads to phosphorylation of BAD. It exerts the separation of antiapoptotic proteins Bcl-XL and Bcl-2 and inhibits the activity of caspase-3. As a result, the apoptosis of cardiac cells is reduced. (e) FGFR1/FGF21/*β*Klotho signaling regulates genes encoding proteins involved in antioxidant pathways: UCP3 and SOD2. It decreases ROS production and oxidative stress in cardiac cells. (f) The main intracellular pathway responsible for FGFR1/FGF21/*β*Klotho action is ERK. PGC1*α* is a transcriptional coactivator of PPAR*γ*, involved in the control of energy metabolism and oxidative stress. The activation of PGC1*α* represses expression of proinflammatory cytokines by targeting NF-*κ*B signaling. It also enhances energy supply by regulation of cardiac lipid metabolism. BAD, Bcl-2-associated death promoter; ER, endoplasmic reticulum; ERK, extracellular signal regulated kinase; FGF21, fibroblast growth factor 21; FGFR1, fibroblast growth factor receptor 1; NF-*κ*B, nuclear factor-kappa-B; PGC1*α*, PPAR*γ* co-activator-1 *α*; PI3K/Akt1, phosphatidylinositol 3-kinase/Akt serine/threonine kinase 1; PPAR*α*, peroxisome proliferator-activated receptor-*α*; ROS, reactive oxygen species; Sirt1, Sirtuin 1; SOD2, superoxide dismutase 2; UCP3, uncoupling protein 3; +, upregulation/increase; -, downregulation/decrease.

The protective role of *β*Klotho in cardiac tissue is based on its interaction with FGF21 as a coreceptor (Figure 3) [43]. The main site of production and release of FGF21 into the blood is considered to be the liver and adipose tissue [87, 88]. Data showed that FGF21 is expressed and released also by cardiomyocytes in animals and humans. Moreover, FGF21 receptor, FGFR1, and *β*-Klotho were present at protein level in cardiac cells [2, 89]. Different cardiac stress stimuli, such as cardiac hypertrophy, transverse aortic constriction, and myocardial infarction, induced expression of FGF21 mRNA in the mouse heart. The isolation of cardiac cells from adult mice confirmed that FGF21 is produced mainly by cardiomyocytes. They showed that isolated neonatal mouse cardiomyocytes secreted FGF21 protein into the cell culture medium. Furthermore, FGF21 level was increased in response to cardiac stress stimuli [89]. The expression of FGF21 was also shown in cardiomyocytes of adult rats. Obese animals had significantly higher level of cardiac FGF21 [45]. Similarly, the expression of cardiac FGF21 was increased after induced endoplasmic reticulum stress and in prooxidative/proinflammatory conditions [90, 91]. An enhanced production of FGF21 in cardiomyocytes was also shown in a mouse model of type 1 diabetes, as well as upon fasting in healthy mice. It indicates a role of FGF21 in energy metabolism under pathological and nonpathological conditions [91, 92]. Interestingly, the expression of FGF21 was found in human heart and it was specifically upregulated in patients at higher cardiovascular risk and during heart failure [2, 90].

Insight into the regulation of FGF21 gene expression in the heart showed that Sirt1-PPAR*α* is a pivotal mechanism [89, 90]. It was shown that the activation of Sirt1 (Sirtuin 1) pathway prevents the development of cardiac hypertrophy and protects cardiac cells from inflammation and metabolic dysregulation. It was mediated through the activation of PPAR*α* (peroxisome proliferator-activated receptor-*α*), a major controller of cardiac lipid metabolism [93]. Interestingly, the heart can be both a target and a source of FGF21. Planavila et al. (2013) demonstrated that Sirt1 induces expression of FGF21 gene in the heart after cardiac stress. They proposed that cardiomyocytes locally generate FGF21 in response to Sirt1. Thus, FGF21 production in an autocrine manner serves as a compensatory mechanism to mitigate the initial damage under cardiac stress [89]. Moreover, an autocrine-acting FGF21 released by cardiomyocytes prevented ROS accumulation and functioned as an antioxidant factor in the heart [90]. In addition to the systemic circulating FGF21, a potential autocrine loop for FGF21 may serve as an endogenous, autoregulatory, cardioprotective pathway.

Some scientists induced the myocardial I/R injury in mice by the occlusion of left anterior descending coronary artery. Interestingly, they tested the signaling mechanism which mediates the FGF21-based myocardial protection. An enhanced production and release of FGF21 from the hepatic cells and adipocytes during cardiac I/R were observed [44]. Data also showed that FGF21 interacted with FGFR1 in cardiomyocytes in the presence of membrane-bound *β*Klotho [44]. Activation of FGFR1/FGF21/*β*Klotho network induced the phosphorylation of cell survival PI3K/Akt1 pathway. Then, Akt1 (Akt serine/threonine kinase 1) phosphorylated the Bcl-XL/Bcl-2-associated death promoter (BAD), and finally it exerted the separation of antiapoptotic proteins Bcl-XL and Bcl-2, which inhibited the activity of caspase-3 and subsequently apoptosis [44, 84, 94]. As a result of this pathway, the reduction of cell death and improvement of myocardial function were observed [44]. This confirms that *β*Klotho is associated with FGFR1/FGF21 signaling in cardiac tissue. Moreover, FGFR1/FGF21 interaction was declined due to suppressed expression of *β*Klotho, and deficiency of FGF21 led to high abundance of apoptotic cells in I/R model [44].

The stimulation of cell survival pathways by FGFR1/FGF21/*β*Klotho complex was also proved in the study of Liang et al. (2017) [95]. Tunicamycin-induced endoplasmic reticulum (ER) stress resulted in the compensatory enhanced level of FGF21 and *β*Klotho in H9c2 rat myoblasts. Moreover, the induction of cell survival ERK1/2 (extracellular signal-regulated kinase 1/2) signaling pathway by FGF21 abolished ER stress-induced injury of cardiomyocytes, increased cell viability, and reduced apoptosis [95]. The activation of ERK1/2 signaling by FGF21 and then cardioprotection was also confirmed in a diabetic mouse model [92]. Similar observations were reported in the study of doxorubicin-induced cardiotoxicity in H9c2 cells. The FGF21 treatment counteracted the oxidative stress, inflammation, and cell death [96].

It was reported that the administration of recombinant FGF21 led to inhibition of inflammation and prooxidative signaling [89, 97]. As previously mentioned, *β*Klotho functions as a coreceptor for FGF21. *β*Klotho and FGFR1 are present in the plasma membrane of cardiomyocytes. The reaction of FGFR1 and *β*-Klotho is necessary for FGF21 sensitivity and ability to activate intracellular signaling pathways [43, 98]. FGFR1/FGF21/*β*Klotho signaling induces cell survival and antioxidative mechanisms and recovery of energy supply in cardiac cells [89, 97]. What is important, the administration of recombinant FGF21 and high expression of *β*-Klotho in cardiac cells improve the function of left ventricular cells. It suggests the beneficial impact of FGF21 and *β*Klotho [45].

The cardioprotective role of FGF21 and *β*Klotho has been confirmed in the hearts of lean and obese rats that were perfused with Langendorff method [45]. After the global ischemia and further reperfusion, the samples of Langendorff coronary effluent, the suspension of isolated cardiomyocytes, and heart tissue were analyzed. Importantly, an infusion of FGF21 protein to rat hearts led to an improvement of myocardial function after injury. Data showed the autocrine/paracrine “positive feedback” loop effect: FGF21 autostimulated the FGF21 production and its secretion from cardiomyocytes in response to obesity and hypoxia [45]. It is possible that the mechanism of cardioprotection is based on an activation of cell survival pathways PI3K/Akt, ERK1/2 (extracellular signal regulated kinase 1/2), and AMPK (AMP-activated protein kinase), via FGFR1/FGF21/*β*Klotho [45, 95]. However, obesity is characterized by FGF21 resistance, increased level of FGF21, and decreased expression of *β*-Klotho in adipose tissue and liver [99, 100]. It has been proposed that TNF-*α* inhibits the expression of *β*-Klotho in adipocytes and impairs FGF21 action. As a result, cells lacking *β*-Klotho were unable to respond to FGF21 [101]. The beneficial effect of FGF21 was impaired in obese rat model. Similarly, obese mice responded poorly to exogenous FGF21 [100]. It was related to the diminished level of *β*Klotho in obese animals, which may account for “FGF21 resistance” and disruption in the FGFR1/FGF21/*β*Klotho signaling [45]. Thus, the proper expression of cardiac *β*Klotho is crucial in cardioprotective effect of FGF21 administration. What is more, FGF21 resistance could also be due to decreased expression of both *β*Klotho and FGF receptors [100]. Tanajak et al. (2016) studied a high‐fat diet‐induced obese, insulin‐resistant rat model. They showed the improvement of cardiometabolic regulation and left ventricular function after long‐term FGF21 administration [102]. It decreased metabolic disturbance, systemic and cardiac oxidative stress, cardiac mitochondrial redox dyshomoeostasis, and structural changes. Moreover, long‐term administration of FGF21 improved FGF21 sensitivity and attenuated resistance of FGF21 and insulin. Importantly, they showed that FGF21-resistant state could be also represented by increased level of plasma FGF21 and decreased cardiac expression of FGFR1, whereas expression of *β*Klotho remained unaltered [102]. These findings indicate a role of FGF21 in heart function under different *β*Klotho background.

FGF21 and *β*Klotho could be involved in the protection of cardiac cells from I/R. The cardioprotective effect of FGF21 was shown in the mouse model of myocardial infarction caused by the permanent ligation of the left anterior descending coronary artery (LAD). The treatment with intramuscularly injected adenoviral vectors expressing FGF21 led to mitigation of left ventricular systolic dysfunction and dilatation after LAD ligation [103]. The main intracellular pathway responsible for FGF21 actions was extracellular signal-regulated kinase (ERK). Planavila et al. (2013) found that treatment of cardiomyocytes in culture with FGF21 activated ERK signaling [89]. The inhibitory action of FGF21 on cardiac hypertrophy and inflammation was associated with the induction of PPAR*γ* co-activator-1 *α* (PGC1*α*). PGC1*α* is a transcriptional coactivator, involved in the control of energy metabolism and oxidative stress in several tissues [89, 104–106]. Importantly, PPAR*γ* represses expression of proinflammatory cytokines by targeting NF-*κ*B signaling [107]. So, the activation of PGC-1*α* axis by FGF21 could be envisioned as a new therapeutic target in metabolic disorders and heart disfunction. Interestingly, in 2015 Planavila et al. revealed new action of FGF21 in prevention of ROS production in cardiac cells [90]. They showed that FGF21 regulates genes encoding proteins involved in antioxidant pathways, uncoupling protein 3 (UCP3) and superoxide dismutase 2 (SOD2). In light of these findings, the administration of FGF21 could inhibit prooxidative/proinflammatory pathways in the heart [90].

The potential impact of these investigations is that FGF21 can improve the function of left ventricular cells and may be considered as a therapeutic agent. It should be emphasized that the cardioprotective action of FGF21 is possible only in the proper expression of FGFR1 and *β*Klotho. Thus, the modulation of FGFR1/FGF21/*β*Klotho signaling pathway could be a novel strategy in cardioprevention.

## 9. Klotho Protein in Cardiac Hypertrophy and Remodeling

Cardiac hypertrophy and remodeling occur in response to various pathogenic stimuli, such as chronic pressure overload, chronic volume overload, or myocardial infarction. Consequently, the pathological alterations of heart structure and function are observed [108, 109]. It is well established that ER stress results in the apoptosis of cardiomyocytes and therefore is involved in the heart remodeling [108, 109]. The study of animal model revealed that Klotho-deficient mice developed an exaggerated pathological cardiac hypertrophy and remodeling after an overstimulation by isoproterenol (ISO) [110]. It was associated with an increased expression of transient receptor potential canonical 6 (TRPC6) channels due to stressful conditions [110]. TRPC, the Ca^2+^-permeable cation channels, are expressed in heart and mediate in the Ca^2+^-dependent signal transduction [110–113]. Their cardiac activity is very low in the physiological state, whereas the stressful factors cause an upregulation of TRPC and abnormal intracellular Ca^2+^-signaling. It is elucidated that TRPC channels, including TRPC1, 3, 4, 5, and 6, promote the cardiac hypertrophy by an activation of calcineurin, nuclear factor of activated T cells (NFAT), the Ca^2+^-dependent hypertrophy-inducing pathway [110–112]. It was shown that administration of soluble Klotho inhibited TRPC6 heart channels and protected hearts from stress-induced cardiac fibrosis [110]. Additionally, the ISO-induced myocardial changes were suppressed in transgenic mice overexpressing Klotho. These novel findings provide compelling evidence that Klotho downregulates TRPC6 channels and rejects the pathological heart alterations [110].

The mechanism of Klotho cardioprotection in myocardial impairment is also based on its antioxidative activity. In 2015 Yang et al. tested an influence of indoxyl sulfate (IS), a uremic toxin, and Klotho protein on the heart function and their association with left ventricular hypertrophy [114]. It is known that IS has the prooxidative and prohypertrophic impact, and its serum level is raised in CKD [114, 115]. Serum Klotho and IS levels were analyzed in patients with chronic kidney disease. It was detected that the low serum Klotho and elevated IS levels were associated with an increased mass of left ventricle [114]. It suggests that both IS and Klotho have an influence on development of LVH in the state of CKD. Similar results were observed in a mouse model. An injection of exogenous IS induced LVH and decreased the expression of renal Klotho in mice. The scientists created also a mouse model of CKD-associated LVH and then supplied animals with the exogenous Klotho protein. Interestingly, the administration of Klotho resulted in the alleviation of LVH [114]. What is more, pretreatment of cultured neonatal rat cardiomyocytes with Klotho led to reduction of IS-related ROS. The research also revealed that ROS overproduction and cardiac hypertrophy arose from an activation of mitogen-activated protein kinase (MAPK) signaling pathway and phosphorylation of MAPK (p38 and ERK1/2) was significantly reduced by exogenous Klotho [114]. Summarizing, Klotho supply leads to the amelioration of LVH due to the inhibition of p38 and ERK1/2 and downregulation of ROS production in cardiomyocytes. For this reason, Klotho protein may initiate the new preventive and therapeutic strategy in left ventricular hypertrophy [114].

In favor of a rationale that supports the compensatory action of Klotho, an association between serum Klotho level and classic risk factors, as well as a clinical history of cardiovascular diseases, has also been investigated. The examination group included 168 older adults with the prior coronary artery disease, myocardial infarction, and stroke [116]. Surprisingly, there was higher concentration of serum Klotho in patients with myocardial infarction in the past. It suggests the compensatory mechanism of Klotho to prevent the development of pathological heart hypertrophy due to the cardiac damage [116]. Interestingly, the compensatory production of Klotho and thus renoprotection has not been noticeable in renal dysfunctions. It is known that Klotho is expressed mainly in the kidney and kidney disorders may contribute to disrupted expression of renal Klotho. The first report of CKD and depletion of Klotho level in the kidney, plasma, and urine was presented in 2011 by Hu et al. [117]. At present, there are a lot of evidences indicating the deficiency of endocrine and renal Klotho due to CKD in humans and in rodent models [118–120]. Downregulation of Klotho is recognized also as an early biomarker for kidney damage and plays a pathogenic role in the progression of CKD [117]. Further, a remarkable reduction of renal Klotho occurred in a swine kidney model after I/R or in rats under sustained circulatory stress [121, 122]. Surprisingly, the level of Klotho mRNA in the kidney was significantly lower in long-term hypertension, diabetes mellitus, and chronic renal failure, but not after acute myocardial infarction [122]. The potential impact of these investigations is compensatory action of Klotho in ischemic heart to prevent cardiac hypertrophy and remodeling. It suggests that renoprotection has not been observed in CKD because of reduced expression of renal Klotho. Notwithstanding, Klotho protein may serve as a potential renoprotective humoral factor by reducing mitochondrial oxidative stress and apoptosis. The replacement of Klotho could be therapeutic in CKD and thus in CVD. An overexpression of Klotho may lead to the compensative amelioration of renal and cardiac injury [54, 123].

It has become clear that hypertensive heart disease is associated with the cardiac fibrosis. Myofibroblasts, the phenotypically transformed fibroblasts, are present in heart due to injury and tissue repair. However, myofibroblasts are also involved in cardiac remodeling and thus contribute to heart disorders [124–126]. In 2016, Liu et al. examined the role of soluble Klotho in the myocardial fibrosis and hypertension [125]. They incubated the cultured myofibroblasts with soluble Klotho, the 130 kDa and 65 kDa isoforms, respectively. As a result of the treatment with 130 kDa Klotho, an intensified proliferation of myofibroblasts and an enhanced synthesis of type I collagen of tested cells were observed. It should be emphasized that raised level of phosphorylated ERK and the MAP kinase was observed [125]. 130 kDa Klotho is a coreceptor for FGF23 and an activation of FGFR pathway leads to the phosphorylation of ERK [7, 125]. So, upregulation of myofibroblasts activity due to Klotho treatment was mediated via the FGFR pathway. Conversely, 65 kDa Klotho suppressed the proliferation and production of collagen in myofibroblasts. These findings were also confirmed in* in vivo* study of mice with hypertension. There was more intensive cardiac interstitial fibrosis in the 130 kDa Klotho differentiation of myofibroblasts, whereas 65 kDa isoform has the antifibrotic effect [125]. This novel finding shows that soluble Klotho isoforms have the opposite regulatory role in cardiac fibrogenesis and open new capabilities in the therapy of heart diseases. Firstly, myofibroblasts appear during myocardial remodeling after cardiac damage and repair injured tissue. They play a role in collagen turnover and scar contraction. Myofibroblasts can also prevent dilatation of the infarct area by maintaining the extracellular matrix in the scar [127]. So, the administration of 130 kDa soluble Klotho enhances the proliferation and differentiation of myofibroblasts and leads to improvement of cardiac function [125]. The stimulatory role of 130 kDa Klotho on cell proliferation has been also reported in other types of cells, like MC3T3.E1 (mouse osteoblastic cell line) and renal PTEC (proximal tubule epithelial cells) [128, 129]. In another hand, the deposition of extracellular matrix at sites remote from the infarct area can lead to cardiac stiffness and the development of heart failure [127]. Therefore, the treatment with 65 kDa Klotho inhibits myofibroblast proliferation and collagen synthesis and could exert antifibrotic effect in cardiac injury [125]. It should be also mentioned that only few experimental works on Klotho account for differential impact of the soluble 130 kDa and 65 kDa isoforms. It was reported that Klotho is expressed in the mouse pancreatic islets of Langerhans with an apparent molecular weight of 65 kDa. A short-form Klotho could bind to the cell membrane and enhance glucose-induced secretion of insulin. It was due to upregulated membrane levels of transient receptor potential V2 (TRPV2), which increases glucose-induced calcium responses [130]. Then, the effect of recombinant short-form Klotho (65 kDa) and full-length Klotho (130 kDa) protein on function of *β*-cell in *β*-islets was assessed. As a result, only short-form Klotho promoted insulin secretion in *β*-islets. The full-length 130 kDa Klotho did not have obvious effect [131]. The production of 65 kDa Klotho was found also in adipose‐derived stem cells (ADSCs), whereas 130 kDa Klotho was not detectable. Interestingly, the deficiency of 65 kDa Klotho suppressed proliferation potential of ADSC and attenuated adipogenic differentiation. It supports the hypothesis of various impacts of Klotho isoforms [132]. However, how soluble 65 kDa Klotho exerts opposite effect to the 130 kDa Klotho in myofibroblasts needs to be studied. The mechanism responsible for the inhibitory and antifibrotic role of 65 kDa isoform remains unknown. Further investigations are needed to unravel the receptors for soluble 65 kDa Klotho. Aditionally, it is difficult to differentiate full-length soluble *α*-Klotho from short fragments, as well as shed from secreted Klotho. This could be the reason why investigators do not make a distinction regarding the treatment with Klotho isoforms and do not account for their suspected differential effect. They mostly describe the administration just of recombinant Klotho. The work of Liu et. al. (2016) highlighted the advantageous usage of soluble isoforms of Klotho as a potential strategy for the development of novel therapeutic interventions in cardiology.

Summing up, there is a rational basis for Klotho administration in the event of heart injury to restrain the further pathological modifications in cardiac tissue. The myocardial protection of Klotho is based on its contribution in the multifarious mechanism. However, it should be considered that the isoforms of Klotho may act as a counter-regulatory factor in the cardiac fibrogenic responses.

## 10. Klotho Protein in Vascular Disorders

It might be important to consider that significant reduction of Klotho expression in vascular wall is associated with the development of artery dysfunction. An expression of endogenous Klotho in the human aortic smooth muscle cells (HA-SMCs) and in the medial layer of renal and epigastric arteries from healthy and CKD patients was described [133]. It was proclaimed that the local deficiency of vascular-derived Klotho led to the calcification of HA-SMCs in procalcific stress conditions [133, 134]. As previously mentioned, Klotho is a coreceptor for FGF23 which induces a negative phosphate and calcium balance. FGFR/FGF23 signaling is necessary to prevent the hyperphosphatemia and thus the calcification [7, 133]. In the state of CKD, Klotho deficiency and elevated level of FGF23 may be developed. Therefore, low level of Klotho is associated with the FGFR/FGF23 resistance, which in turn dismisses the anticalcific effects of FGF23 [133, 134]. In addition, given that FGF23 suppresses the synthesis of biologically active 1,25-dihydroxyvitamin D_3_, it was proved that vitamin D receptor (VDR) ligands render the cardiovascular survival benefits [20, 22, 133]. An activation of VDR resulted in the restoration of Klotho expression and hence the FGFR response. So, it provided the protection against the proliferation of HA-SMCs and pathological calcific changes in vascular wall. For this reason, vascular Klotho can act as an endogenous inhibitor of calcification and mediates between FGF23 and vascular cells [133].

A large number of studies over the last decade have greatly enriched our knowledge of the antioxidative activity of Klotho and its correlation with an endothelium function. In 2016, Richter et al. analyzed an influence of synthesis of NO mediated by FGF23 and oxidative stress on human coronary artery endothelial cells (HCAEC) [135]. As mentioned before, the binding affinity of FGF23 to its receptors, FGFR1c, 3c, and 4, is enhanced by Klotho protein [7, 14]. Data showed an enhanced expression of membrane-bound Klotho and FGFR1, 2, and 4 in HCAEC. Moreover, Klotho and FGFR1 were expressed also in human coronary arteries. FGF23 stimulation in HCAEC resulted in the activation of FGFR1 signaling, as well as in the shedding of soluble Klotho* via* ADAM17 protease [135]. Afterwards, the FGFR1/FGF23/Klotho complex caused an activation of the Akt/eNOS signaling pathway in HCAEC. Finally, eNOS engendered the production of NO, which is a known vasodilative factor improving the function of endothelial cells [135].

It is also suggested that an activity of NADPH oxidase 2 (Nox2) leads to the generation of ROS, while MnSOD and catalase detoxify ROS. The investigation showed that FGF23 increases the expression of Nox2 and antioxidant proteins [135]. Essentially, the positive effect of FGF23 was associated with Klotho protein. The balance between formation and degradation of ROS stimulated by FGF23 was maintained only in the presence of Klotho. What is important, in absence of Klotho the production of ROS was enhanced, while the ROS detoxification and NO synthesis were blunted. Thus, Klotho deficiency and FGF23 overexpression led to imbalance between Klotho and FGF23 and then to the oxidative stress and endothelial complications [135].

Reduction of oxidative stress may contribute to the development of effective treatment strategies for atherosclerosis. In 2017 Yao et al. analyzed an association between Klotho and atherogenesis in an ox-LDL-induced endothelial cell injury model with human umbilical vein endothelial cells (HUVECs) [136]. HUVECs were treated with the different concentrations of recombinant human Klotho and oxidized low-density lipoprotein (ox-LDL). Ox-LDL is recognized as a proinflammatory and atherogenic factor. Then, intracellular production of ROS and NO and the activity of SOD were measured. Interestingly, Klotho averted the cytotoxicity of ox-LDL and improved cell viability. It was associated with lower production of ROS and induction of SOD activity in HUVECs [136]. Data also indicated that Klotho protein ameliorated the ox-LDL-induced endothelial dysfunction* via* an activation of PI3K/Akt/eNOS pathway. Consequently, the enhancement of eNOS activity and production of NO were observed [136].

Klotho treatment also reduced the expression of lectin-like oxidized low-density lipoprotein receptor-1 (LOX-1). LOX-1 is a major ox-LDL receptor in endothelial cells, which is a crucial factor in the pathogenesis of atherosclerosis [136]. Thus, the inhibition of the LOX-1 pathway by Klotho restrains the inflammatory response and atherogenesis [136].

To conclude, the presence of Klotho protein in vascular wall is necessary for the functional amelioration of vasculature and protects against the procalcific processes.

## 11. Conclusion

There are many well documented evidences indicating the antioxidative and antiapoptotic activity of Klotho protein. It is also well established that Klotho plays an important role in the prevention of cardiovascular diseases, particularly in the maintaining of appropriate cardiac and vascular function. Klotho is involved in mechanisms of defense against the development of heart hypertrophy and remodeling, as well as the vascular calcification and atherogenesis. Many studies showed that Klotho deficiency or* KLOTHO* gene polymorphism can be the risk factors for the most prevalent cardiovascular diseases. Thus, the regulation of serum Klotho and FGFs level and their expression in cardiomyocytes could be essential for the cell metabolism, proper heart function, and protection in some disorders. Given the apparent importance of Klotho, it may be considered as a novel vital factor in the ischemic heart injury, such as myocardial infarction. Conclusively, it appears plausible that an activity of Klotho could be protective in a damaged myocardial tissue and open new path for the treatment of cardiovascular diseases.

## References

[1] Nichols M., Townsend N., Scarborough P., Rayner M. (2014). Cardiovascular disease in Europe 2014: epidemiological update. *European Heart Journal*.

[2] Corsetti G., Pasini E., Scarabelli T. M. (2016). Decreased expression of Klotho in cardiac atria biopsy samples from patients at higher risk of atherosclerotic cardiovascular disease. *Journal of Geriatric Cardiology*.

[3] Kuro-o M., Matsumura Y., Aizawa H. (1997). Mutation of the mouse klotho gene leads to a syndrome resembling ageing. *Nature*.

[4] Shiraki-Iida T., Aizawa H., Matsumura Y. (1998). Structure of the mouse klotho gene and its two transcripts encoding membrane and secreted protein 1. *FEBS Letters*.

[5] Matsumura Y., Aizawa H., Shiraki-Iida T., Nagai R., Kuro-O M., Nabeshima Y.-I. (1998). Identification of the human klotho gene and its two transcripts encoding membrane and secreted klotho protein. *Biochemical and Biophysical Research Communications*.

[6] Li S.-A., Watanabe M., Yamada H., Nagai A., Kinuta M., Takei K. (2004). Immunohistochemical localization of Klotho protein in brain, kidney, and reproductive organs of mice. *Cell Structure and Function*.

[7] Kim J., Hwang K., Park K., Kong I. D., Cha S. (2015). Biological Role of Anti-aging Protein Klotho. *Journal of Lifestyle Medicine*.

[8] Chen C.-D., Podvin S., Gillespie E., Leeman S. E., Abraham C. R. (2007). Insulin stimulates the cleavage and release of the extracellular domain of Klotho by ADAM10 and ADAM17. *Proceedings of the National Acadamy of Sciences of the United States of America*.

[9] Imura A., Iwano A., Tohyama O. (2004). Secreted Klotho protein in sera and CSF: implication for post-translational cleavage in release of Klotho protein from cell membrane. *FEBS Letters*.

[10] Bloch L., Sineshchekova O., Reichenbach D. (2009). Klotho is a substrate for *α*-, *β*- and *γ*-secretase. *FEBS Letters*.

[11] Doi S., Zou Y., Togao O. (2011). Klotho inhibits transforming growth factor-*β*1 (TGF-*β*1) signaling and suppresses renal fibrosis and cancer metastasis in mice. *The Journal of Biological Chemistry*.

[12] Cararo-Lopes M. M., Mazucanti C. H. Y., Scavone C., Kawamoto E. M., Berwick D. C. (2017). The relevance of *α*-KLOTHO to the central nervous system: Some key questions. *Ageing Research Reviews*.

[13] Szymczak A., Forma E. (2012). Struktura i funkcje bialka Klotho. *Folia Medica Lodz*.

[14] Hu M. C., Shiizaki K., Kuro-O M., Moe O. W. (2013). Fibroblast growth factor 23 and klotho: physiology and pathophysiology of an endocrine network of mineral metabolism. *Annual Review of Physiology*.

[15] Xu Y., Sun Z. (2015). Molecular basis of klotho: From gene to function in aging. *Endocrine Reviews*.

[16] Xiao N., Zhang Y., Zheng Q., Gu J. (2004). Klotho is a serum factor related to human aging. *Chinese Medical Journal*.

[17] Kuro-o M. (2011). Klotho and the aging process. *The Korean Journal of Internal Medicine*.

[18] Yamazaki Y., Imura A., Urakawa I. (2010). Establishment of sandwich ELISA for soluble alpha-Klotho measurement: age-dependent change of soluble alpha-Klotho levels in healthy subjects. *Biochemical and Biophysical Research Communications*.

[19] Kato Y., Arakawa E., Kinoshita S. (2000). Establishment of the anti-klotho monoclonal antibodies and detection of klotho protein in kidneys. *Biochemical and Biophysical Research Communications*.

[20] Tsujikawa H., Kurotaki Y., Fujimori T., Fukuda K., Nabeshima Y.-I. (2003). Klotho, a gene related to a syndrome resembling human premature aging, functions in a negative regulatory circuit of vitamin D endocrine system. *Molecular Endocrinology*.

[21] Andrukhova O., Smorodchenko A., Egerbacher M. (2014). FGF23 promotes renal calcium reabsorption through the TRPV5 channel. *EMBO Journal*.

[22] Abousaab A., Warsi J., Salker M. S., Lang F. (2016). *β*-Klotho as a Negative Regulator of the Peptide Transporters PEPT1 and PEPT2. *Cellular Physiology and Biochemistry*.

[23] Ben-Dov I. Z., Galitzer H., Lavi-Moshayoff V. (2007). The parathyroid is a target organ for FGF23 in rats. *The Journal of Clinical Investigation*.

[24] Tohyama O., Imura A., Iwano A. (2004). Klotho is a novel beta-glucuronidase capable of hydrolyzing steroid beta-glucuronides. *The Journal of Biological Chemistry*.

[25] Tan S., Smith E. R., Holt S. G., Hewitson T. D., Toussaint N. D. (2017). Soluble klotho may be a marker of phosphate reabsorption. *Clinical Kidney Journal*.

[26] Hoenderop J. G. J., Nilius B., Bindels R. J. M. (2005). Calcium absorption across epithelia. *Physiological Reviews*.

[27] Lee W.-S., Hebert S. C. (1995). ROMK inwardly rectifying ATP-sensitive K+ channel. I. Expression in rat distal nephron segments. *American Journal of Physiology - Renal Fluid and Electrolyte Physiology*.

[28] Dong K., Yan Q., Lu M. (2016). Romk1 knockout mice do not produce bartter phenotype but exhibit impaired K excretion. *The Journal of Biological Chemistry*.

[29] Welling P. A., Ho K. (2009). A comprehensive guide to the ROMK potassium channel: form and function in health and disease. *American Journal of Physiology-Renal Physiology*.

[30] Bhide G. P., Colley K. J. (2017). Sialylation of N-glycans: mechanism, cellular compartmentalization and function. *Histochemistry and Cell Biology*.

[31] Stanley P., Schachter H., Taniguchi N., Varki A., Cummings R. D., Esko J. D. (2009). N-Glycans. *Essentials of Glycobiology*.

[32] Cha S.-K., Hu M.-C., Kurosu H., Kuro-O M., Moe O., Huang C.-L. (2009). Regulation of renal outer medullary potassium channel and renal K + excretion by Klotho. *Molecular Pharmacology*.

[33] Cha S.-K., Ortega B., Kurosu H., Rosenblatt K. P., Kuro M., Huang C.-L. (2008). Removal of sialic acid involving Klotho causes cell-surface retention of TRPV5 channel via binding to galectin-1. *Proceedings of the National Acadamy of Sciences of the United States of America*.

[34] Camby I., Le Mercier M., Lefranc F., Kiss R. (2006). Galectin-1: a small protein with major functions. *Glycobiology*.

[35] Wolf M. T. F., An S.-W., Nie M., Bal M. S., Huang C.-L. (2014). Klotho up-regulates renal calcium channel transient receptor potential vanilloid 5 (TRPV5) by intra- And extracellular N-glycosylation-dependent mechanisms. *The Journal of Biological Chemistry*.

[36] Leunissen E. H. P., Nair A. V., Büll C. (2013). The epithelial calcium channel TRPV5 is regulated differentially by klotho and sialidase. *The Journal of Biological Chemistry*.

[37] Dalton G. D., Xie J., An S., Huang C. (2017). New Insights into the Mechanism of Action of Soluble Klotho. *Frontiers in Endocrinology*.

[38] DeFronzo R. A., Felig P., Ferrannini E., Wahren J. (1980). Effect of graded doses of insulin on splanchnic and peripheral potassium metabolism in man. *American Journal of Physiology-Endocrinology and Metabolism*.

[39] Kurosu H., Yamamoto M., Clark J. D. (2005). Suppression of aging in mice by the hormone klotho. *Science*.

[40] Utsugi T., Ohno T., Ohyama Y. (2000). Decreased insulin production and increased insulin sensitivity in the klotho mutant mouse, a novel animal model for human aging. *Metabolism*.

[41] Hu M. C., Shi M., Zhang J. (2015). Renal production, uptake, and handling of circulating *α*Klotho. *Journal of the American Society of Nephrology*.

[42] Hu M. C., Shi M., Zhang J. (2010). Klotho: A novel phosphaturic substance acting as an autocrine enzyme in the renal proximal tubule. *FASEB Journal : Official Publication of the Federation of American Societies for Experimental Biology*.

[43] Ogawa Y., Kurosu H., Yamamoto M. (2007). *β*Klotho is required for metabolic activity of fibroblast growth factor 21. *Proceedings of the National Acadamy of Sciences of the United States of America*.

[44] Liu S. Q., Roberts D., Kharitonenkov A. (2013). Endocrine protection of ischemic myocardium by FGF21 from the liver and adipose tissue. *Scientific Reports*.

[45] Patel V., Adya R., Chen J. (2014). Novel insights into the cardio-protective effects of FGF21 in lean and obese rat hearts. *PLoS ONE*.

[46] Lan T., Morgan D. A., Rahmouni K. (2017). FGF19, FGF21, and an FGFR1/*β*-Klotho-activating antibody act on the nervous system to regulate body weight and glycemia. *Cell Metabolism*.

[47] Yamamoto M., Clark J. D., Pastor J. V. (2005). Regulation of oxidative stress by the anti-aging hormone klotho. *The Journal of Biological Chemistry*.

[48] Bartke A. (2006). Long-lived Klotho mice: new insights into the roles of IGF-1 and insulin in aging. *Trends in Endocrinology & Metabolism*.

[49] Thurston R. D., Larmonier C. B., Majewski P. M. (2010). Downregulation of aging-related Klotho gene in experimental colitis: the role of TNF and IFN-*γ*. *Gastroenterology*.

[50] Zhang X., Yalcin S., Lee D.-F. (2011). FOXO1 is an essential regulator of pluripotency in human embryonic stem cells. *Nature Cell Biology*.

[51] Takenaka T., Kobori H., Inoue T. (2017). [op.4b.02] klotho supplementation attenuates blood pressure and oxidative stress in diabetes. *Journal of Hypertension*.

[52] Lim S. W., Jin L., Luo K. (2017). Klotho enhances FoxO3-mediated manganese superoxide dismutase expression by negatively regulating PI3K/AKT pathway during tacrolimus-induced oxidative stress. *Cell Death & Disease*.

[53] Mitobe M., Yoshida T., Sugiura H., Shirota S., Tsuchiya K., Nihei H. (2005). Oxidative stress decreases klotho expression in a mouse kidney cell line. *Nephron Experimental Nephrology*.

[54] Sugiura H., Yoshida T., Tsuchiya K. (2005). Klotho reduces apoptosis in experimental ischaemic acute renal failure. *Nephrology Dialysis Transplantation *.

[55] Oh H. J., Nam B. Y., Lee M. J. (2015). Decreased circulating Klotho levels in patients undergoing dialysis and relationship to oxidative stress and inflammation. *Journal of the International Seociety for Peritoneal Dialysis*.

[56] Elghoroury E. A., Fadel F. I., Elshamaa M. F. (2018). Klotho G-395A gene polymorphism: impact on progression of end-stage renal disease and development of cardiovascular complications in children on dialysis. *Pediatric Nephrology*.

[57] Kuro-o M. (2009). Klotho and aging. *Biochimica et Biophysica Acta (BBA) - General Subjects*.

[58] Arking D. E., Krebsova A., Macek M. (2002). Association of human aging with a functional variant of klotho. *Proceedings of the National Acadamy of Sciences of the United States of America*.

[59] Deary I. J., Harris S. E., Fox H. C. (2005). KLOTHO genotype and cognitive ability in childhood and old age in the same individuals. *Neuroscience Letters*.

[60] Mengel-From J., Soerensen M., Nygaard M., McGue M., Christensen K., Christiansen L. (2016). Genetic variants in KLOTHO associate with cognitive function in the oldest old group. *The Journals of Gerontology. Series A*.

[61] Marchelek-Myśliwiec M., Różański J., Ogrodowczyk A. (2016). The association of the Klotho polymorphism rs9536314 with parameters of calcium-phosphate metabolism in patients on long-term hemodialysis. *Renal Failure*.

[62] Arking D. E., Becker D. M., Yanek L. R. (2003). KLOTHO allele status and the risk of early-onset occult coronary artery disease. *American Journal of Human Genetics*.

[63] Arking D. E., Atzmon G., Arking A., Barzilai N., Dietz H. C. (2005). Association between a functional variant of the KLOTHO gene and high-density lipoprotein cholesterol, blood pressure, stroke, and longevity. *Circulation Research*.

[64] Oguro R., Kamide K., Kokubo Y. (2010). Association of carotid atherosclerosis with genetic polymorphisms of the klotho gene in patients with hypertension. *Geriatrics & Gerontology International*.

[65] Telci D., Dogan A. U., Ozbek E. (2011). KLOTHO gene polymorphism of G395A is associated with kidney stones. *American Journal of Nephrology*.

[66] Kawano K.-I., Ogata N., Chiano M. (2002). Klotho gene polymorphisms associated with bone density of aged postmenopausal women. *Journal of Bone and Mineral Research*.

[67] Imamura A., Okumura K., Ogawa Y. (2006). Klotho gene polymorphism may be a genetic risk factor for atherosclerotic coronary artery disease but not for vasospastic angina in Japanese. *Clinica Chimica Acta*.

[68] Rhee E. J., Oh K. W., Yun E. J. (2006). Relationship between polymorphisms G395A in promoter and C1818T in exon 4 of the KLOTHO gene with glucose metabolism and cardiovascular risk factors in Korean women. *Journal of Endocrinological Investigation*.

[69] Semba R. D., Cappola A. R., Sun K. (2011). Plasma klotho and cardiovascular disease in adults. *Journal of the American Geriatrics Society*.

[70] Keles N., Caliskan M., Dogan B. (2015). Low serum level of Klotho is an early predictor of atherosclerosis. *The Tohoku Journal of Experimental Medicine*.

[71] Keusch G., Boengler K., Schulz R. (2008). Cardioprotection: nitric oxide, protein kinases, and mitochondria. *Circulation*.

[72] Mungrue I. N., Gros R., You X. (2002). Cardiomyocyte overexpression of iNOS in mice results in peroxynitrite generation, heart block, and sudden death. *The Journal of Clinical Investigation*.

[73] Förstermann U., Sessa W. C. (2012). Nitric oxide synthases: regulation and function. *European Heart Journal*.

[74] Dias A. S., Porawski M., Alonso M., Marroni N., Collado P. S., González-Gallego J. (2005). Quercetin decreases oxidative stress, NF-*κ*B activation, and iNOS overexpression in liver of streptozotocin-induced diabetic rats. *Journal of Nutrition*.

[75] Krause M., Heck T. G., Bittencourt A., Scomazzon S. P., Newsholme P., Curi R. (2015). The Chaperone Balance Hypothesis: The Importance of the Extracellular to Intracellular HSP70 Ratio to Inflammation-Driven Type 2 Diabetes, the Effect of Exercise, and the Implications for Clinical Management. *Mediators of Inflammation*.

[76] Drew D. A., Katz R., Kritchevsky S. (2017). Association between soluble klotho and change in kidney function: The health aging and body composition study. *Journal of the American Society of Nephrology*.

[77] Hu M. C., Shi M., Gillings N. (2017). Recombinant *α*-Klotho may be prophylactic and therapeutic for acute to chronic kidney disease progression and uremic cardiomyopathy. *Kidney International*.

[78] Marçais C., Maucort-Boulch D., Drai J. (2017). Circulating klotho associates with cardiovascular morbidity and mortality during hemodialysis. *The Journal of Clinical Endocrinology & Metabolism*.

[79] Ene-Iordache B., Perico N., Bikbov B. (2016). Chronic kidney disease and cardiovascular risk in six regions of the world (ISN-KDDC): A cross-sectional study. *The Lancet Global Health*.

[80] Takeshita K., Fujimori T., Kurotaki Y. (2004). Sinoatrial Node Dysfunction and Early Unexpected Death of Mice with a Defect of klotho Gene Expression. *Circulation*.

[81] Zhou H., Li H., Shi M. (2018). Protective Effect of Klotho against Ischemic Brain Injury Is Associated with Inhibition of RIG-I/NF-*κ*B Signaling. *Frontiers in Pharmacology*.

[82] Shi M., Flores B., Gillings N. (2016). *α*klotho mitigates progression of aki to ckd through activation of autophagy. *Journal of the American Society of Nephrology*.

[83] Neyra J. A., Hu M. C. (2017). Potential application of klotho in human chronic kidney disease. *Bone*.

[84] Hui H., Zhai Y., Ao L. (2017). Klotho suppresses the inflammatory responses and ameliorates cardiac dysfunction in aging endotoxemic mice. *Oncotarget *.

[85] Guo Y., Zhuang X., Huang Z. (2018). Klotho protects the heart from hyperglycemia-induced injury by inactivating ROS and NF-*κ*B-mediated inflammation both in vitro and in vivo. *Biochimica et Biophysica Acta (BBA) - Molecular Basis of Disease*.

[86] Nguyen T., Nioi P., Pickett C. B. (2009). The Nrf2-antioxidant response element signaling pathway and its activation by oxidative stress. *The Journal of Biological Chemistry*.

[87] Hondares E., Iglesias R., Giralt A. (2011). Thermogenic activation induces FGF21 expression and release in brown adipose tissue. *The Journal of Biological Chemistry*.

[88] Badman M. K., Pissios P., Kennedy A. R., Koukos G., Flier J. S., Maratos-Flier E. (2007). Hepatic fibroblast growth factor 21 is regulated by PPAR*α* and is a key mediator of hepatic lipid metabolism in ketotic states. *Cell Metabolism*.

[89] Planavila A., Redondo I., Hondares E. (2013). Fibroblast growth factor 21 protects against cardiac hypertrophy in mice. *Nature Communications*.

[90] Planavila A., Redondo-Angulo I., Ribas F. (2015). Fibroblast growth factor 21 protects the heart from oxidative stress. *Cardiovascular Research*.

[91] Brahma M. K., Adam R. C., Pollak N. M. (2014). Fibroblast growth factor 21 is induced upon cardiac stress and alters cardiac lipid homeostasis. *Journal of Lipid Research*.

[92] Zhang C., Huang Z., Gu J. (2015). Fibroblast growth factor 21 protects the heart from apoptosis in a diabetic mouse model via extracellular signal-regulated kinase 1/2-dependent signalling pathway. *Diabetologia*.

[93] Planavila A., Iglesias R., Giralt M., Villarroya F. (2011). Sirt1 acts in association with PPAR*α* to protect the heart from hypertrophy, metabolic dysregulation, and inflammation. *Cardiovascular Research*.

[94] Roy S. K., Srivastava R. K., Shankar S. (2010). Inhibition of PI3K/AKT and MAPK/ERK pathways causes activation of FOXO transcription factor, leading to cell cycle arrest and apoptosis in pancreatic cancer. *Journal of Molecular Signaling*.

[95] Liang P., Zhong L., Gong L. (2017). Fibroblast growth factor 21 protects rat cardiomyocytes from endoplasmic reticulum stress by promoting the fibroblast growth factor receptor 1-extracellular signal-regulated kinase 1/2 signaling pathway. *International Journal of Molecular Medicine*.

[96] Wang S., Wang Y., Zhang Z., Liu Q., Gu J. (2017). Cardioprotective effects of fibroblast growth factor 21 against doxorubicin-induced toxicity via the SIRT1/LKB1/AMPK pathway. *Cell Death & Disease*.

[97] Cong W.-T., Ling J., Tian H.-S. (2013). Proteomic study on the protective mechanism of fibroblast growth factor 21 to ischemia-reperfusion injury. *Canadian Journal of Physiology and Pharmacology*.

[98] Adams A. C., Cheng C. C., Coskun T., Kharitonenkov A. (2012). FGF21 Requires *β*klotho to Act In Vivo. *PLoS ONE*.

[99] Gallego-Escuredo J. M., Gómez-Ambrosi J., Catalan V. (2015). Opposite alterations in FGF21 and FGF19 levels and disturbed expression of the receptor machinery for endocrine FGFs in obese patients. *International Journal of Obesity*.

[100] Fisher F. M., Chui P. C., Antonellis P. J. (2010). Obesity is a fibroblast growth factor 21 (FGF21)-resistant state. *Diabetes*.

[101] Díaz-Delfín J., Hondares E., Iglesias R., Giralt M., Caelles C., Villarroya F. (2012). TNF-*α* represses *β*-klotho expression and impairs FGF21 action in adipose cells: Involvement of JNK1 in the FGF21 pathway. *Endocrinology*.

[102] Tanajak P., Sa-Nguanmoo P., Wang X. (2016). Fibroblast growth factor 21 (FGF21) therapy attenuates left ventricular dysfunction and metabolic disturbance by improving FGF21 sensitivity, cardiac mitochondrial redox homoeostasis and structural changes in pre-diabetic rats. *Acta Physiologica*.

[103] Joki Y., Ohashi K., Yuasa D. (2015). FGF21 attenuates pathological myocardial remodeling following myocardial infarction through the adiponectin-dependent mechanism. *Biochemical and Biophysical Research Communications*.

[104] Chau M. D. L., Gao J., Yang Q., Wu Z., Gromada J. (2010). Fibroblast growth factor 21 regulates energy metabolism by activating the AMPK-SIRT1-PGC-1*α* pathway. *Proceedings of the National Acadamy of Sciences of the United States of America*.

[105] Lehman J. J., Kelly D. P. (2002). Transcriptional activation of energy metabolic switches in the developing and hypertrophied heart. *Clinical and Experimental Pharmacology and Physiology*.

[106] Ventura-Clapier R., Garnier A., Veksler V. (2008). Transcriptional control of mitochondrial biogenesis: the central role of PGC-1*α*. *Cardiovascular Research*.

[107] Eisele P. S., Salatino S., Sobek J., Hottiger M. O., Handschin C. (2013). The peroxisome proliferator-activated receptor *γ* coactivator 1*α*/*β* (PGC-1) coactivators repress the transcriptional activity of NF-*κ*B in skeletal muscle cells. *The Journal of Biological Chemistry*.

[108] Song S., Gao P., Xiao H., Xu Y., Si L. Y. (2013). Klotho suppresses cardiomyocyte apoptosis in mice with stress-induced cardiac injury via downregulation of endoplasmic reticulum stress. *PLoS ONE*.

[109] Song S., Si L.-Y. (2015). Klotho ameliorated isoproterenol-induced pathological changes in cardiomyocytes via the regulation of oxidative stress. *Life Sciences*.

[110] Xie J., Cha S.-K., An S.-W., Kuro-O M., Birnbaumer L., Huang C.-L. (2012). Cardioprotection by Klotho through downregulation of TRPC6 channels in the mouse heart. *Nature Communications*.

[111] Wu X., Eder P., Chang B., Molkentin J. D. (2010). TRPC channels are necessary mediators of pathologic cardiac hypertrophy. *Proceedings of the National Acadamy of Sciences of the United States of America*.

[112] Bush E. W., Hood D. B., Papst P. J. (2006). Canonical transient receptor potential channels promote cardiomyocyte hypertrophy through activation of calcineurin signaling. *The Journal of Biological Chemistry*.

[113] Sabourin J., Robin E., Raddatz E. (2011). A key role of TRPC channels in the regulation of electromechanical activity of the developing heart. *Cardiovascular Research*.

[114] Yang K., Wang C., Nie L. (2015). Klotho protects against indoxyl sulphate-induced myocardial hypertrophy. *Journal of the American Society of Nephrology*.

[115] Barreto F. C., Barreto D. V., Liabeuf S. (2009). Serum indoxyl sulfate is associated with vascular disease and mortality in chronic kidney disease patients. *Clinical Journal of the American Society of Nephrology*.

[116] Paula R. S., Souza V. C., Machado-Silva W. (2016). Serum Klotho (but not haplotypes) associate with the post-myocardial infarction status of older adults. *Clinics*.

[117] Hu M. C., Shi M., Zhang J. (2011). Klotho deficiency causes vascular calcification in chronic kidney disease. *Journal of the American Society of Nephrology*.

[118] Zhao Y., Banerjee S., Dey N. (2011). Klotho depletion contributes to increased inflammation in kidney of the db/db mouse model of diabetes via RelA (serine)536 phosphorylation. *Diabetes*.

[119] Hu M.-C., Kuro-o M., Moe O. W. (2010). Klotho and kidney disease. *Journal of Nephrology*.

[120] Levey A. S., Atkins R., Coresh J. (2007). Chronic kidney disease as a global public health problem: approaches and initiatives—a position statement from kidney disease improving global outcomes. *Kidney International*.

[121] Castellano G., Intini A., Stasi A. (2016). Complement modulation of anti-aging factor klotho in ischemia/reperfusion injury and delayed graft function. *American Journal of Transplantation*.

[122] Aizawa H., Saito Y., Nakamura T. (1998). Downregulation of the klotho gene in the kidney under sustained circulatory stress in rats. *Biochemical and Biophysical Research Communications*.

[123] Haruna Y., Kashihara N., Satoh M. (2007). Amelioration of progressive renal injury by genetic manipulation of Klotho gene. *Proceedings of the National Acadamy of Sciences of the United States of America*.

[124] Weber K. T. (2000). Fibrosis and hypertensive heart disease. *Current Opinion in Cardiology*.

[125] Liu X., Chen Y., Mccoy C. W. (2016). Differential regulatory role of soluble klothos on cardiac fibrogenesis in hypertension. *American Journal of Hypertension*.

[126] Weber K. T. (2004). Fibrosis in hypertensive heart disease: Focus on cardiac fibroblasts. *Journal of Hypertension*.

[127] Van Den Borne S. W. M., Diez J., Blankesteijn W. M., Verjans J., Hofstra L., Narula J. (2010). Myocardial remodeling after infarction: the role of myofibroblasts. *Nature Reviews Cardiology*.

[128] Shalhoub V., Ward S. C., Sun B. (2011). Fibroblast growth factor 23 (FGF23) and *α*-klotho stimulate osteoblastic MC3T3.E1 cell proliferation and inhibit mineralization. *Calcified Tissue International*.

[129] Medici D., Razzaque M. S., DeLuca S. (2008). FGF-23-Klotho signaling stimulates proliferation and prevents vitamin D-induced apoptosis. *The Journal of Cell Biology*.

[130] Lin Y., Sun Z. (2012). Antiaging gene Klotho enhances glucose-induced insulin secretion by up-regulating plasma membrane levels of TRPV2 in MIN6 *β*-cells. *Endocrinology*.

[131] Lin Y., Sun Z. (2015). In vivo pancreatic *β*-cell-specific expression of antiaging gene Klotho: a novel approach for preserving *β*-cells in type 2 diabetes. *Diabetes*.

[132] Fan J., Sun Z. (2016). The antiaging gene klotho regulates proliferation and differentiation of adipose-derived stem cells. *Stem Cells*.

[133] Lim K., Lu T., Molostvov G. (2012). Vascular klotho deficiency potentiates the development of human artery calcification and mediates resistance to fibroblast growth factor 23. *Circulation*.

[134] Olauson H., Vervloet M. G., Cozzolino M., Massy Z. A., Torres P. U., Larsson T. E. (2014). New insights into the FGF23-Klotho axis. *Seminars in Nephrology*.

[135] Richter B., Haller J., Haffner D., Leifheit-Nestler M. (2016). Klotho modulates FGF23-mediated NO synthesis and oxidative stress in human coronary artery endothelial cells. *Pflügers Archiv - European Journal of Physiology*.

[136] Yao Y., Wang Y., Zhang Y., Liu C. (2017). Klotho ameliorates oxidized low density lipoprotein (ox-LDL)-induced oxidative stress via regulating LOX-1 and PI3K/Akt/eNOS pathways. *Lipids in Health and Disease*.

