# Corrigendum to “The Biological Role of Klotho Protein in the Development of Cardiovascular Diseases”

**DOI:** 10.1155/2020/1463925

**Published:** 2020-09-29

**Authors:** Agnieszka Olejnik, Aleksandra Franczak, Anna Krzywonos-Zawadzka, Marta Kałużna-Oleksy, Iwona Bil-Lula

**Affiliations:** ^1^Department of Medical Laboratory Diagnostics, Division of Clinical Chemistry, Wroclaw Medical University, 50-556 Wroclaw, Poland; ^2^Department of Cardiology, University Hospital of Lord's Transfiguration, Poznan University of Medical Sciences, 61-848 Poznan, Poland

In the article titled “The Biological Role of Klotho Protein in the Development of Cardiovascular Diseases” [1], the grant number was incorrect in the “Acknowledgements” section. The corrected section appears below:

## References

[1] Olejnik A., Franczak A., Krzywonos-Zawadzka A., Kałużna-Oleksy M., Bil-Lula I. (2018). The Biological Role of Klotho Protein in the Development of Cardiovascular Diseases. *BioMed Research International*.

